# Novel Application of Transcutaneous Electrical Stimulation for Ophthalmoplegia in Miller Fisher Syndrome: A Case Report

**DOI:** 10.3390/healthcare13233154

**Published:** 2025-12-03

**Authors:** Ying-Chi Huang, Fred Yi-Shueh Chen

**Affiliations:** 1 Department of Physical Medicine and Rehabilitation, Taipei Medical University Hospital, Taipei 11031, Taiwan; 213022@h.tmu.edu.tw; 2 Department of Physical Medicine and Rehabilitation, Ditmanson Medical Foundation Chia-Yi Christian Hospital, Chia-Yi 60002, Taiwan

**Keywords:** Guillain–Barré syndrome, Miller Fisher syndrome, ophthalmoplegia, transcutaneous electrical nerve stimulation

## Abstract

We report the case of a 62-year-old woman with Miller Fisher syndrome (MFS) whose severe bilateral ophthalmoplegia showed no improvement after four weeks of standard care consisting of intravenous immunoglobulin and physiotherapy. High-frequency transcutaneous electrical nerve stimulation (TENS) was applied over the bilateral sternocleidomastoid muscles and immediately followed by eye-movement exercises. Within three days, ocular motility began to improve, and after one month, only minimal left-eye abduction lag remained. Such neuromodulation of TENS might act on central and peripheral tiers of the oculomotor system and may have been temporally associated with faster recovery than expected in the natural course of the disease compared to the median three-month interval. This dramatic, time-associated improvement highlights the novelty of cervical TENS as a potential accelerator for recovery in MFS-related ophthalmoplegia. To the best of our knowledge, research involving the use of TENS in MFS-related ophthalmoplegia is limited, and our case demonstrates the feasibility and safety of TENS for faster recovery.

## 1. Introduction

Guillain–Barré syndrome (GBS) is the most common acute paralytic neuropathy, affecting around 100,000 people annually worldwide [1]. The annual incidence in Western countries ranges from 0.8 to 1.9 cases per 100,000 people; risk of GBS increases with age, and diagnosis is slightly more frequent in males [1,2,3]. GBS is typically triggered by an infection or other immune stimulation, leading to an aberrant autoimmune response targeting the peripheral nervous system. Clinically, it presents as rapidly progressive bilateral limb weakness, often with sensory and cranial nerve involvement, reaching its peak deficit within 2–4 weeks [1,3]. Reflexes are usually decreased or absent. Diagnosis is largely based on clinical patterns. It is supported by cytoalbuminological dissociation, where cerebrospinal fluid examination typically shows an elevated protein level with a normal white cell count [2]. Nerve conduction studies also aid in diagnosis and subtype classification [1]. GBS is an umbrella term covering several recognizable variants with distinct features. The main subtypes include Acute Inflammatory Demyelinating Polyneuropathy (AIDP) and Acute Motor Axonal Neuropathy (AMAN) [1,2,3].

Miller Fisher syndrome (MFS) is another well-defined variant characterized by ophthalmoplegia, ataxia, and areflexia [4,5,6]. Ophthalmoparesis, typically bilateral, often progresses over one to two weeks, and severe ataxia can lead to an inability to walk without support [7]. Additional signs may include ptosis, facial nerve palsy, and distal paresthesia. The etiology of MFS is immune-mediated, as symptoms frequently follow antecedent infections, particularly upper respiratory or gastrointestinal infections, with Campylobacter jejuni and Haemophilus influenzae being common culprits [5,6]. The underlying mechanism is “molecular mimicry,” where antibodies, primarily anti-GQ1b IgG, are produced against pathogen components that resemble the GQ1b ganglioside. This ganglioside is highly expressed in oculomotor nerves, deep cerebellar nuclei, and neuromuscular spindles, explaining the characteristic symptoms. Anti-GQ1b IgG antibodies are detected in over 90% of MFS patients [8].

MFS is uncommon, with an annual incidence of less than 1 in a million. It accounts for a small percentage of GBS cases and is more prevalent in Asian countries [9]. The prognosis for MFS is generally excellent, with spontaneous and complete recovery being the natural course [5]. Most patients fully recover from ophthalmoplegia and ataxia within months, although areflexia can persist for longer [10]. Treatment typically involves immunomodulating therapies such as intravenous immunoglobulin (IVIG) or plasmapheresis [5,6,10]. While IVIG may slightly accelerate symptom amelioration, these treatments do not significantly alter final recovery outcomes due to the disease’s favorable natural course. Although ophthalmoplegia in patients with MFS is typically self-limited and associated with a good prognosis, recovery still often takes weeks to months [5,10]. Our objective is to demonstrate the feasibility of applying TENS to the bilateral neck muscles in patients with MFS-related ophthalmoplegia. This case study has been reported in line with the CARE guidelines [11].

## 2. Patient Information

A 62-year-old woman with a remote history of breast cancer surgery arrived at the emergency department on Hospital Day 1 with rapidly progressive binocular diplopia, blurred vision, and unsteady gait. Examination revealed almost complete horizontal and marked vertical gaze limitation in both eyes, mild truncal and limb ataxia, and generalized areflexia, with preserved cognition and pupillary reflexes. Cranial CT was unremarkable, and CSF examination on Day 1 demonstrated normal protein and cell counts. Nerve conduction studies performed on Days 2 and 9 demonstrated absent F-waves and H-reflexes with reduced CMAP amplitudes in multiple motor nerves but without conduction block. Clinical findings strongly suggested Miller Fisher syndrome (MFS), so intravenous immunoglobulin (total 2 g kg^−1^) was administered over Days 1–5, arresting further neurological decline. On Hospital Day 6, brain MRI was performed, showing no evidence of brainstem stroke or alternative structural causes of ophthalmoplegia, supporting the peripheral pattern suggested clinically. Throughout this period, the patient reported substantial functional difficulty with balance and visual tasks, consistent with the disabling nature of early MFS. The overall diagnostic reasoning incorporated the classical triad of ophthalmoplegia, ataxia, and areflexia, the exclusion of central causes on imaging, and the expected evolution of early ancillary tests, which together formed a coherent clinical picture despite initially non-diagnostic CSF and electrophysiologic findings.

Conventional physiotherapy began on Day 2, including a structured ten-minute eye movement rehabilitation program. This program consisted of horizontal and vertical smooth pursuit, saccadic refixation between two spaced targets, brief eccentric gaze-holding, near-target convergence tasks, and full-range sweeps across the eight cardinal positions, with progressive increases in movement amplitude and speed as tolerated; however, the ophthalmoplegia remained fixed. An ophthalmology review on Day 12 confirmed bilateral oculomotor, trochlear, and abducens palsies with lagophthalmos, and ocular lubricants were prescribed, but extra-ocular motility (EOM) deficits persisted at −4 in all directions. Extraocular movements were systematically graded using a −4 to +4 scale, with −4 indicating no movement and 0 representing full motility. Because ocular function had plateaued, cervical TENS applied bilaterally over the mid-belly of the sternocleidomastoid muscles was initiated on Day 26, followed by the eye movement rehabilitation program. The parameters are shown in Table 1. No additional medications, repeat IVIG, plasmapheresis, or new rehabilitation modalities were introduced during or after the TENS intervention.

**Figure 1:**
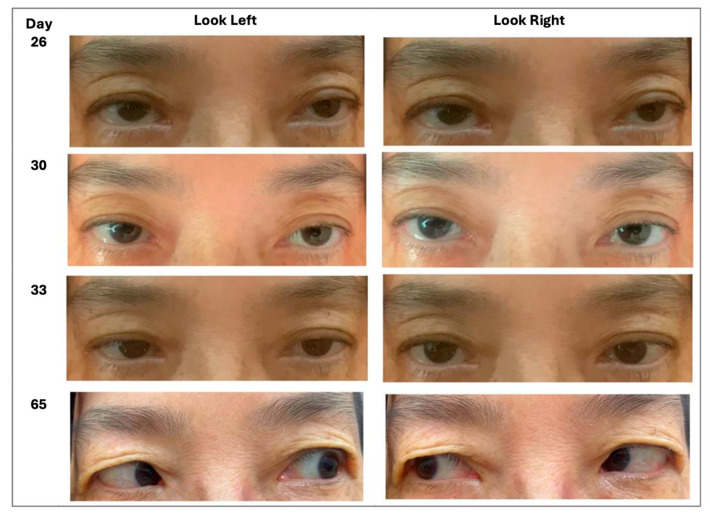
Progressive improvement in horizontal gaze corresponding to the clinical timeline (Day 26 = start of TENS; Days 29–33 = early improvement; Day 65 = follow-up).

From that point, the patient’s recovery was distinctly stepwise, as depicted in Figure 1. Three days after the first TENS session (Hospital Day 29), right-eye adduction improved to −3 and the caregiver noticed smoother horizontal tracking. The next morning (Hospital Day 30), formal assessment confirmed a comparable, though slightly smaller, gain in the left eye, together with modest improvements in abduction and vertical excursion. By the fourth day of stimulation (Day 31), the horizontal range had widened further: right-eye adduction had advanced to −2, left-eye adduction to −3, and the right-eye abduction deficit had eased to −4. Two days later, on Day 33, adduction was symmetrical at −2 in both eyes, abduction remained at −4, and diplopia was now confined to extreme left gaze. No adverse effects were recorded.

The patient was discharged on Day 35 without diplopia in primary gaze and was enrolled in outpatient rehabilitation. She continued cervical TENS three times per week for one month, maintaining the combined protocol with ongoing eye movement training. One month later (Day 65), follow-up photographs showed full elevation, depression, and adduction in both eyes and only a faint residual deficit in left-eye abduction (−1). She reported no diplopia during ordinary visual tasks and was cleared to resume all premorbid activities, illustrating a sustained improvement during the combined rehabilitation program.

## 3. Discussion

Functional electrical stimulation (FES) and neuromuscular electrical stimulation (NMES), along with TENS, have been explored as therapeutic modalities in GBS patients, with emerging evidence regarding their safety and efficacy. A pilot study evaluating NMES and muscle fiber stimulation in the early, severe phase of GBS found them to be safe and feasible as an adjunct therapy, with no treatment-related adverse effects recorded aside from initial discomfort that was managed by adjusting stimulation intensity [12,13,14,15,16]. Stimulation for one hour four to five times per week was deemed feasible even in an acute hospital setting [14]. With this understanding regarding safety and feasibility, we decided to take a combined modality approach to our patient’s treatment after a lack of improvement with IVIG.

In chronic GBS, FES combined with task-oriented grasp training successfully improved fine motor skills, and these improvements were maintained over time without continued stimulation, indicating the effectiveness of this treatment in terms of promoting meaningful motor gains [12]. This approach was particularly effective in addressing disuse atrophies when lower motoneurons remained intact. Furthermore, TENS has a long history of use in pain management and is considered a valuable adjunct for patient comfort, having demonstrated the ability to significantly decrease pain in the back and lower extremities of GBS patients [13,16]. Beyond pain relief, TENS has shown anti-inflammatory and regenerative effects, enhancing the therapeutic outcome of conventional treatments in a case of post-COVID-19 GBS and acute transverse myelitis overlap syndrome, leading to significant reductions in neuropathic pain, motor, and sensory deficits and a regression of neurophysiological changes [16]. In an attempt to alleviate our patient’s severe bilateral ophthalmoplegia, we incorporated TENS into our treatment for enhanced motor gains.

A review of the literature showed that TENS applied to neck muscles addresses various conditions. For neck pain, when used as part of conventional physiotherapy (e.g., with hot packs), TENS contributed to significant improvements in pain, disability, and isometric neck muscle strength [15]. In cases of dysphonia, low-frequency TENS on neck and submandibular muscles significantly decreases musculoskeletal pain in the neck, shoulders, upper/lower back, and masseter. These effects are attributed to comfortable muscle contractions, increased blood flow, and the release of opioid peptides, which can also influence distant body parts.

MFS carries an excellent prognosis, with spontaneous and complete recovery being the natural course. Ophthalmoplegia, a core symptom, typically presents early and often bilaterally, progressing within 1–2 weeks [5,6]. While treatments like IVIG may slightly accelerate initial amelioration (with a median of around 12–13 days to the start of improvement), they do not significantly alter the time to complete disappearance (with a median of up to 3 months) [10]. Most patients achieve full resolution, often within months [5,8], with 96% being symptom-free within one year [10]. Studies indicate that the lateral rectus muscle (LR) is the most involved extraocular muscle (EOM) and the last to recover, frequently resulting in esotropia as the most common pattern of ocular deviation. In this study, incomplete ophthalmoplegia (IO) was more prevalent than complete ophthalmoplegia (CO) [7].

TENS delivered to the sternocleidomastoid muscles offers a biologically plausible means of accelerating recovery from Miller Fisher-related ophthalmoplegia by acting on both central and peripheral tiers of the oculomotor system [17,18,19,20]. At the peripheral level, high-frequency sensory TENS floods the cervical dorsal horns with large-diameter group I/II afferents, closing spinal gate circuitry and unleashing descending opioid pathways that ease nociception and inhibit α-motoneuron overactivity [8,15,16,21,22]. The resulting relaxation of suboccipital, peri-orbital, and cervical musculature reduces passive drag on the globe, lowers vergence effort, and enables the patient to execute residual eye movements with less mechanical resistance. In parallel, experimental work in neuropathic and demyelinating models shows that repeated transcutaneous stimulation can heighten nerve growth factor expression, enlarge axon caliber, promote remyelination, and curb pro-inflammatory cytokines [16,19,20]. Perhaps these changes could create a more permissive milieu for repair of the anti-GQ1b-injured cranial nerve terminals characteristic of MFS.

Centrally, cervical proprioceptive inflow ascends through the cuneate nucleus to multisensory hubs [18,20] in the thalamus, parieto-insular vestibular cortex, and cerebellar nodulus, all of which project to the frontal and supplementary eye fields [18,20]. Functional imaging and electrophysiological studies in spatial neglect reveal that neck muscle TENS shortens abnormally prolonged visual evoked potential latencies and restores contralesional spatial maps, demonstrating an ability to recalibrate large-scale networks that integrate eye, head, and trunk positions [18,19,20]. Such neuromodulation is particularly relevant because MFS, though classically defined as a peripheral neuropathy, often exhibits transient cerebellar ataxia and brainstem involvement, implying that central oculomotor circuits are susceptible to adaptive plasticity once an adequate proprioceptive trigger is provided [12,20,21,23]. The temporal profile observed in our patient, where a three-day inflection occurred after a four-week plateau and near-complete ocular recovery within one month, is compatible with the hypothesis that such mechanisms might interact to influence the trajectory of recovery from ophthalmoplegia. By first relaxing cervical and peri-orbital tone, TENS may unmask latent motor capacity. The subsequent eye-movement exercises, performed immediately, then capitalize on heightened cortical excitability to reinforce the newly available range, creating a closed loop of afferent priming and task-specific training [12,21]. Given the uncontrolled design of this case, the observed acceleration in ocular motility cannot be attributed definitively to TENS. Several alternative explanations warrant consideration. The improvement may have resulted from the natural course of MFS, which is characterized by spontaneous and often rapid remission. The structured eye movement training program administered immediately after TENS may also have played an independent role, or the combination of TENS-mediated sensory priming and task-specific training may have produced a synergistic effect. Furthermore, a delayed or cumulative therapeutic response to intravenous immunoglobulin cannot be excluded. Because multiple concurrent or sequential factors may have contributed to the improvement, the association between TENS and recovery should be regarded as temporal rather than causal.

This report has several limitations that constrain its interpretability. Anti-GQ1b antibody testing, which provides strong diagnostic support for MFS, was not available in this case. Electrophysiological studies were obtained only during the early disease phase, when characteristic demyelinating features may be absent. Early ancillary testing in MFS is frequently nondiagnostic, as cerebrospinal fluid protein often remains normal within the first week and typical electrophysiologic abnormalities may not appear until later in the disease course [2]. Published electrodiagnostic series report that only approximately 50–65% of patients with GBS-related variants, including MFS, demonstrate abnormal nerve conduction findings within the first one to two weeks, with absent late responses often preceding more definitive demyelinating changes [24].

Although spontaneous remission is the rule in MFS, the dramatic, time-associated improvement seen here suggests that cervical TENS could serve as a low-risk, inexpensive adjunct when ocular deficits stagnate after initial immunotherapy [6,10]. The lack of a natural history comparator and absent objective measures, such as visual evoked potentials, quantitative eye-tracking, or prism cover testing, means that both the effect size and proposed mechanisms remain speculative. All ocular assessments were conducted by a single non-blinded evaluator. Although several physiological mechanisms have been proposed to explain how cervical TENS might influence oculomotor recovery, these remain theoretical and cannot be established from a single uncontrolled clinical observation. Rigorous sham-controlled trials with quantitative eye-tracking, vestibular testing, and neuroimaging are needed to confirm efficacy, optimize dosing, and identify patients most likely to benefit from the addition of TENS. Until such data emerge, clinicians may reasonably consider a carefully monitored trial of TENS in patients whose diplopia persists beyond the expected recovery window, provided no contraindications exist.

## 4. Conclusions

This case demonstrates that adding cervical TENS to standard care was temporally associated with the patient’s recovery from ophthalmoplegia. Given the treatment’s favorable safety profile, ease of application, and minimal cost, a carefully monitored trial of TENS may be justified in MFS patients whose ocular deficits plateau after initial immunotherapy. Future studies should incorporate sham controls, quantitative eye-tracking, and neurophysiological measures to confirm causality, refine stimulation parameters, and determine which patient subgroups stand to benefit most from this novel rehabilitative approach.

## Figures and Tables

**Table 1:** Summary of cervical TENS parameters.

Parameter	Specification
Device Model	Likon 320
Waveform	Sensory-level TENS
Frequency	100 Hz
Pulse Width	100 µs
Intensity	Strong but comfortable sensory-level stimulation (without visible contraction)
Electrode Size	4 × 4 cm
Electrode Placement	Bilateral mid-belly sternocleidomastoid muscles
Session Duration	10 minutes
Impatient Schedule	Daily, Day 26–35
Outpatient Schedule	3 times/week for 1 month
Total Sessions	7 inpatient + 12 outpatient

## Data Availability

The data presented in this study are available on request from the corresponding author due to privacy restriction.
